# Impact of antenatal screening for Chlamydia and Gonorrhea on pregnancy outcomes in South African women

**DOI:** 10.1186/s12879-026-13967-3

**Published:** 2026-07-21

**Authors:** Dorothy Chiwoniso Nyemba, Alex de Voux, Rufaro Mvududu, Remco P. H. Peters, Sinead Delany-Moretlwe, Leigh F. Johnson, Thomas J. Coates, Landon Myer, Dvora Leah Joseph Davey

**Affiliations:** 1 Division of Epidemiology and Biostatistics, School of Public Health, University of Cape Town, Cape Town, South Africa; 2 Wits RHI, University of the Witwatersrand, Johannesburg, South Africa; 3 Division of Medical Microbiology, University of Cape Town, Cape Town, South Africa; 4 Department of Epidemiology, Fielding School of Public Health, University of California Los Angeles, Los Angeles, CA USA; 5 Centre for Integrated Data & Epidemiological Research, School of Public Health, University of Cape Town, Cape Town, South Africa; 6 Division of Infectious Diseases, David Geffen School of Medicine, University of California Los Angeles, Los Angeles, CA USA

**Keywords:** Antenatal care, Chlamydia, Gonorrhea, Screening, Pregnancy outcome

## Abstract

**Background:**

We evaluated the impact of diagnosing and treating curable STIs during pregnancy on adverse pregnancy outcomes in Cape Town, South Africa.

**Methods:**

Eligible women *≥* 15 years, without HIV, attending first antenatal care (ANC) visit before 28 weeks gestation, received diagnostic testing at enrolment and either same-day or follow-up treatment for *Chlamydia trachomatis* (CT) and *Neisseria gonorrhoeae* (NG) using either on-site GeneXpert or laboratory-based testing with TaqMan qPCR assays. We assessed prevalence of CT/NG, time to treatment and the frequency of poor pregnancy outcomes, individually and combined including pregnancy loss (miscarriage [< 20weeks] or stillbirth [≥ 20weeks]) and perinatal outcomes (low birthweight [< 2500 g], preterm birth [< 37weeks], small for gestational age [< 10th percentile] and neonatal death [< 7days]) among women with or without CT/NG at study enrolment. Logistic regression models evaluated the impact of time to CT/NG treatment on the frequency of individual and combined poor pregnancy outcomes, adjusting for maternal characteristics.

**Results:**

Among 1237 pregnant women, the prevalence of CT/NG at first ANC visit was 28% (24% CT, 8% NG). Of those diagnosed with CT/NG (*n* = 345), 54% received immediate treatment(< 7 days), while 34% experienced delayed treatment (median time to treatment 28 days (interquartile range: 15–57)) and 12% were untreated during pregnancy. Among women with CT/NG, the frequency of miscarriage (12%) and stillbirth (9%) were highest among women with untreated CT/NG, lower among delayed treatment (4% and 3%), and lowest among immediate treatment (1% and 2%), respectively. Among women with CT/NG at enrolment, poor pregnancy outcome was 28%, 23%, and 29% for immediate, delayed, and no treatment, respectively; 22% among women with no CT/NG at enrolment (overall χ² test, *p* = 0.05). Untreated CT/NG were associated with increased odds of pregnancy loss (adjusted odds ratio (aOR); 3.27, 95% CI: 1.09–9.80) compared to CT/NG with immediate treatment and compared to no CT/NG at study enrolment (aOR; 5.05, 95% CI: 2.23–11.43). Multivariable analysis showed no significant difference in poor pregnancy outcome by time to CT/NG treatment.

**Conclusion:**

Untreated CT/NG during pregnancy increased the odds of experiencing a miscarriage or stillbirth. These findings suggest potential benefits of STI screening using diagnostic tests leading to the timely treatment of curable STIs in pregnancy, and randomized trials are urgently needed to clarify these effects.

**Clinical trial number:**

Not applicable.

**Supplementary Information:**

The online version contains supplementary material available at 10.1186/s12879-026-13967-3.

## Background

Curable sexually transmitted infections (STIs) during pregnancy are associated with adverse pregnancy outcomes, including miscarriage, stillbirth, preterm birth (PTB), low birthweight (LBW) and several secondary life-threatening conditions in surviving neonates [1–5]. Although syphilis screening and treatment is included in routine antenatal care [6], more evidence is needed to understand the impact of screening, diagnosis and treatment for other curable STIs such as *Chlamydia trachomatis* (CT) and *Neisseria gonorrhoeae* (NG) during pregnancy on adverse pregnancy outcomes.

Untreated maternal CT infection is associated with adverse obstetric outcomes such as fetal loss, premature rupture of membranes, preterm labour and PTB in most but not all studies [2, 7, 8]. CT infections during pregnancy can also result in vertical transmission of the infection to the baby during delivery and may lead to conjunctivitis and pneumonia in newborns [1, 8]. Untreated maternal NG infection is associated with LBW [4, 9], small for gestational age (SGA) [2], PTB, premature rupture of membranes and septic abortion [4, 10]. Vertical transmission of maternal NG infection to newborns can cause neonatal conjunctivitis [4, 9]. While untreated maternal CT or NG infections can lead to genital tract inflammation, which has been shown to increase mucosal susceptibility to HIV acquisition and, in the presence of maternal HIV, may enhance the risk of vertical HIV transmission [9, 11]. The burden of untreated maternal CT or NG infections on maternal and child health in low- and middle-income countries (LMICs) is substantial. For example, these infections have been linked to an increased risk of adverse pregnancy outcomes, with estimated odds ratios of 1.46 for stillbirth, 1.46 for PTB, and 1.48 for LBW [12]. This is particularly concerning given that these poor pregnancy outcomes remain the leading cause of mortality among children under five years of age [13].

Despite the demonstrated threat to maternal and child health posed by high prevalence of asymptomatic CT or NG infections during pregnancy [14–16], routine antenatal testing is not mandatory by World Health Organization (WHO), nor is it part of the standard of care in antenatal settings in most LMICs [17]. Evidence regarding the effectiveness of aetiological screening and treatment of curable STIs during pregnancy in reducing adverse pregnancy and birth outcomes remains inconsistent. A randomized trial of presumptive STI therapy during pregnancy in Uganda demonstrated that therapy with azithromycin 1000 mg, cefixime 400 mg and metronidazole 2 g reduced the incidence of LBW and PTB [18]. While the recent WANTAIM trial, which focused on point-of-care (POC) testing and treatment for CT, NG, *T. vaginalis*, and bacterial vaginosis during pregnancy, did not show an overall decrease in PTB or LBW, among women with NG in the same day testing and treatment group a 53% lower risk of PTB or LBW was observed [19]. The Maduo study in Botswana found a reduction in PTB and LBW prevalence in the POC screening and treatment group compared to the standard care group (11% vs. 16%), although this difference was not statistically significant [20]. In a non-randomized prospective cohort study of women living with HIV in South Africa, aetiological testing for CT, NG and *T. vaginalis* was effective in reducing the high prevalence of these curable STIs among pregnant women compared to syndromic management [21]. However, no significant difference was observed between the groups in rates of PTB or LBW. In all these studies, pregnant women who tested positive for STIs received ceftriaxone and azithromycin for CT and NG, and metronidazole for *T. vaginalis* and bacterial vaginosis (22–24).

There is increasing recognition of the limitations of syndromic management in women who may have asymptomatic infection [14–16], and the WHO guidelines now encourage aetiological testing where resources allow. Nevertheless, aetiological screening has not been routinely integrated into antenatal care for CT or NG in most LMICs primarily due to two key barriers: limited resources for diagnostic tests [22, 23], compounded by limited and inconsistent evidence demonstrating the benefits of screening and treating these infections during pregnancy to reduce adverse pregnancy outcomes [4, 7]. New molecular tests for curable STIs such as GeneXpert for CT and NG are being introduced [24]. Evidence shows that POC and near-POC testing can substantially shorten time to treatment compared with centralized laboratory models. Previous research in the HVTN 702 trial, participants diagnosed using rapid POC testing initiated treatment approximately 39-fold faster than those tested through standard laboratory pathways with delayed result return [25]. Similarly, a study in antenatal care in Cape Town reported a mean time to STI results of 0 days with POC testing versus 26 days with laboratory testing, and a corresponding reduction in time to treatment (3 vs. 38 days). Among women diagnosed with an STI, 28% in the laboratory group did not return for treatment compared with only 4% in the POC group [26]. However, the effectiveness of near-POC platforms such as GeneXpert which often have longer processing times than true rapid tests depends on patients’ willingness and ability to wait for results. A pilot study among adolescents and young adults in Cape Town found that only about one-third of participants waited for same-day results when CT/NG testing was conducted on-site using GeneXpert with an approximately 90-minute turnaround time [27].

In this study, we assessed prevalence of CT/NG, time to treatment of the diagnosed CT/NG, the frequency of poor pregnancy outcomes and evaluated the impact of time to treatment of diagnosed CT/NG on poor pregnancy outcomes in a cohort of women without HIV.

## Methods

### Study design and setting

The PrEP in Pregnant and Postpartum women (PrEP-PP) study was a prospective cohort in which consenting pregnant girls and women (age ≥ 15 years) without HIV were enrolled at their first antenatal clinic (ANC) visit or at ANC follow up, as previously described [28]. Briefly, the pregnant girls and women had to have a confirmed pregnancy, be without HIV based on a 4th generation antigen/antibody combination HIV test, intend to stay in Cape Town through study period and have no contraindications to PrEP. Participants were followed monthly during pregnancy. Participants were recruited from a primary healthcare facility located in the urban township of Gugulethu, Cape Town, South Africa. The facility serves a population of approximately 400,000 people, primarily of low socioeconomic status and in 2019, the HIV prevalence among pregnant women who used ANC services at the Gugulethu Midwife Obstetrics Unit was around 30% [29].

### Study participants and procedure

The cohort of women without HIV in our study provides a valuable opportunity to examine the effects of CT and NG on pregnancy outcomes in the absence of HIV-related inflammation. Previous studies have found that pregnant women living with HIV have higher levels of inflammatory markers than those without HIV, and persistent inflammation is linked to an increased risk of adverse pregnancy outcomes, particularly PTB and LBW [30, 31]. This study allows for a clearer understanding of the specific impact these curable STIs have on maternal and infant health, without the confounding inflammatory processes caused by HIV infection.

Consecutive pregnant adolescent girls and women were recruited into the study from August 2019 to October 2021. The PrEP-PP study was approved by the Human Research Ethics Committee at the University of Cape Town (#297/2018) and by the University of California, Los Angeles Institutional Review Board (IRB#18-001622). Written informed consent was provided by all participants in English or the local language, isiXhosa. Eligible, consenting participants received HIV prevention counselling from trained study staff, offered PrEP and trained study interviewers conducted surveys using standardized questionnaires at enrolment. At enrolment, women were offered diagnostic testing for CT/NG through self-collected lateral vaginal wall swab specimens using on-site GeneXpert Vaginal/Endocervical Specimen Collection kits (Cepheid, Sunnyvale, CA) from August 2019 until November 2020 [28]. Women were to receive results and treatment before leaving the clinic. From December 2020 until October 2021, Cepheid was unable to provide the STI testing kits, and the self-collected vaginal swabs were sent to the Department of Pathology (University of Cape Town) for CT and NG testing using TaqMan™ Vaginal Microbiota assays as per manufacturer instructions (Thermo Fisher Scientific, South Africa). Test results were returned to study staff and women with positive results were immediately contacted via telephone, SMS or at the next study visit (1-month after the baseline visit) if telephonic contact was not possible. All women presenting with symptoms, as well as those diagnosed with CT/NG were offered treatment in accordance with the South African national guidelines [32]. Women diagnosed with CT/NG were given partner notification slips specifying the infection to support partner management. Testing for CT and NG was conducted only at enrolment, and no test of cure was performed. As part of standard care, women were also screened and treated for syphilis, however, these data were not captured in the study database.

### Data collection and management

Data on maternal demographics, pregnancy history and healthcare information was collected in a standardized questionnaire attached as supplementary material. Socio-economic status (SES) was determined using an asset-based score that combine maternal education and employment status, the type of housing and household belongings. Pregnancy and obstetric outcomes were abstracted from obstetric and maternity case records at delivery facilities by study clinician. Gestational age was estimated based on the date of the last menstrual period or ultrasound dating recorded in the maternity case records at first antenatal care visit. Birth anthropometrics were abstracted from the child Road to Health Booklet at the 7-day postpartum visit where mother-infant pairs were evaluated by study teams. Miscarriage was defined as non-induced pregnancy loss before 20 weeks of gestation. Stillbirth was defined as delivery of a baby with no sign of life at or after 20 weeks gestation. Early neonatal death was defined as death of a new-born between zero and seven days after birth. Preterm birth (PTB) was defined as birth before completion of 37 weeks gestation. Low birthweight (LBW) was defined as an infant birthweight less than 2500 g. Small for gestational age (SGA) was defined as infants whose birth weight falls below the 10th percentile for their gestational age, as determined using Intergrowth 21st software. Miscarriage, stillbirth, and early neonatal death were assessed through participant self-report and abstraction from obstetric and maternity case records. PTB, LBW and SGA were determined using data extracted from the child’s Road to Health Booklet and clinical assessments by obstetric staff at delivery facilities. In addition, we used WHO guidelines to categorize adverse pregnancy outcomes based on gestational age, timing of the first antenatal care visit, and abstracted pregnancy outcome [33]. STI testing, results, and treatment outcome data were captured and documented by the study’s data coordinators at enrolment and during follow-up visits. PrEP initiation and use data were also recorded by study’s data coordinators at enrolment and during follow-up visits and PrEP use was defined as use or no use during pregnancy. For this study, we restricted analysis to participants who had a CT/NG test at enrolment (on-site GeneXpert or laboratory-based) and available pregnancy and birth outcomes.

### Exposures and outcomes

**Fig. 1:**
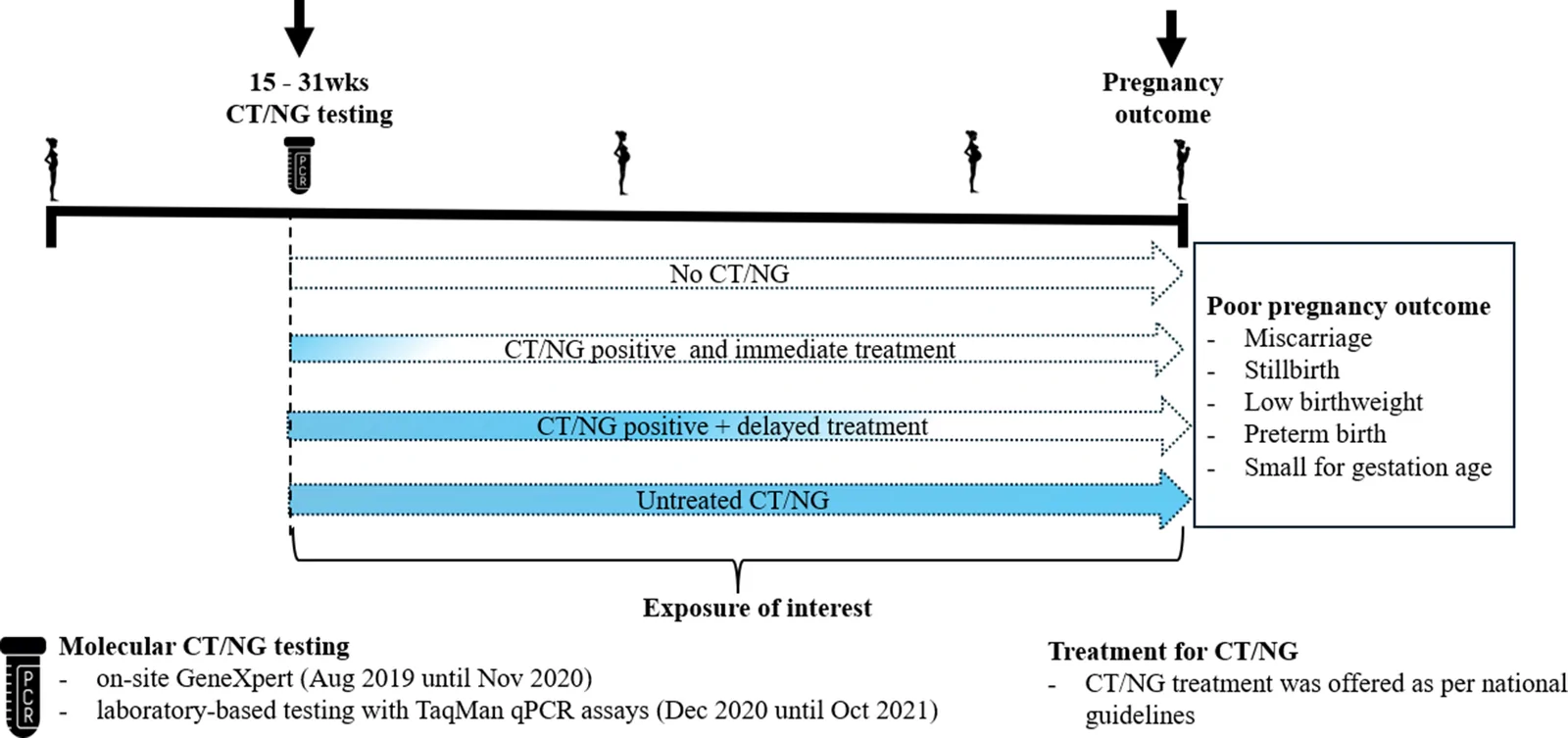
Timeline of exposure and primary outcome of interest

The primary outcome of interest was a poor pregnancy outcome, defined as having at least one adverse outcome including pregnancy loss (miscarriage, stillbirth) and perinatal outcome (PTB, LBW, SGA or early neonatal death) (Fig. 1**)**. The primary exposure was time to treatment following a positive diagnosis of CT or NG as shown in Fig. 1. Given the use of both near-POC GeneXpert and laboratory-based diagnostic testing with differing turnaround times, we defined immediate CT/NG treatment as treatment received within a week from day of diagnosis to accommodate expected operational delays associated with laboratory testing and patient recall while still reflecting clinically timely management of the infections. CT/NG diagnosis with delayed treatment was defined as treatment received a week or more after day of diagnosis, but during pregnancy. Untreated were defined as participants with CT/NG diagnosed but with no treatment documented as received throughout the pregnancy. In a secondary analysis, we classified participants into four exposure groups: CT/NG‑negative, CT/NG‑positive with immediate treatment, CT/NG‑positive with delayed treatment, and CT/NG‑positive without treatment.

### Statistical analysis

The sample size requirements were determined based on a pilot study assessing the feasibility and acceptability of oral PrEP among pregnant women [28]. A sample size of 1,200 was sufficiently powered (> 80%) to detect statistically significant differences of 5% in the proportion of any adverse outcome between time to treatment groups. For all statistical tests, a significance level α of 0.05 was used. Additionally, an a priori decision was made to include maternal age, calendar period, gravidity, socioeconomic and marital status informed by our previous analysis and literature.

Maternal characteristics were compared by CT/NG status at enrolment using the Wilcoxon test, χ2 test or Fisher exact test as appropriate. We fitted logistic regressions to estimate odds ratio and 95% confidence interval (CI) for all association analyses. All the models adjusted for a priori confounders including maternal age, calendar period, gravidity, socioeconomic and relationship status. The calendar period for STI testing was categorized as ‘pre-COVID-19 lockdown’ for tests conducted before 15 March 2020, and ‘during COVID-19 lockdown’ for tests conducted between 15 March 2020 and 31 October 2021. We performed sensitivity analyses. First, for poor pregnancy outcome among women who had enrolled in the study early in pregnancy with gestational age less than 20 weeks. Second, we restricted our analysis to pregnant women who initiated HIV PrEP during pregnancy. Third, we stratified delayed treatment group into categories based on time to treatment (8–14 days, 15–28 days, > 28 days). All analyses were performed using STATA 18 (StataCorp, College Station, TX, USA).

## Results

### Baseline characteristics

Between August 2019 and October 2021, 1306 pregnant women without HIV and not using oral PrEP were enrolled. Of these, 96% (1261/1306) had a CT/NG test at study enrolment and a pregnancy outcome recorded (six participants did not receive CT/NG testing and 39 were lost to follow-up with no recorded pregnancy outcome). Excluding two women who had elective termination of pregnancy, seven who seroconverted for HIV and fifteen multiple live births, 1237 mother-infant were eligible for pregnancy and birth outcome analysis. The median age of all pregnant women was 26 years (interquartile range [IQR]: 22–31) and the median gestational age at enrolment was 22 weeks (IQR: 15–31) (Table 1). Most women (66%) had had at least one prior pregnancy, half of the women had completed grade 12, most were unemployed (65%) and 33% were defined as of low socioeconomic status. Almost all women (97%) reported being sexually active during pregnancy, with 3% (*n* = 43) reporting more than one partner in the past 3 months and 70% reported condomless sex at last sexual intercourse. 70% of women reported being in a concordant HIV-negative relationship and 54% of the women did not perceive themselves to be at risk for HIV infection, (Table 1). Compared to pregnant women without CT/NG infection, those with a positive CT/NG diagnosis at enrolment were more likely to be younger, primigravid and not married or cohabiting, (Table 1). No significant differences were observed in baseline characteristics between participants receiving GeneXpert testing and those receiving laboratory-based CT/NG testing, indicating that the shift in testing modality across COVID-19 periods is unlikely to have introduced bias (Table S1).

*Diagnosis of CT and NG at study enrolment.*

Of the 1237 pregnant women included in this analysis, more than half (57%, 699/1237) received CT/NG testing using on-site GeneXpert and 43% (538/1237) received laboratory-based testing. Overall, 28% (345/1237) were diagnosed with CT and/or NG at enrolment with 56% (193/345) from on-site GeneXpert testing and 44% (152/345) from laboratory-based testing, (Table 1).

### Treatment of CT and or NG during pregnancy

**Fig. 2:**
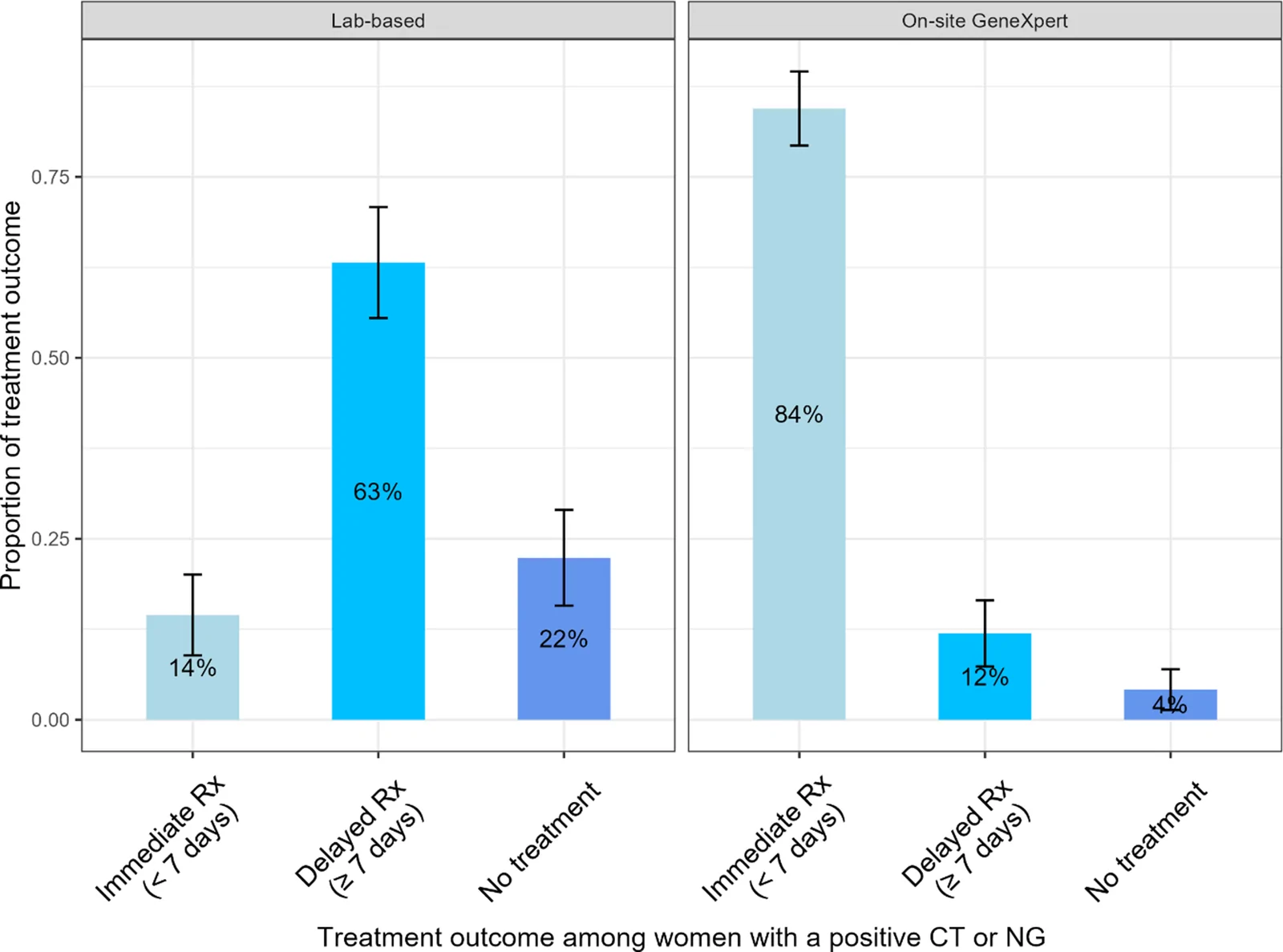
Treatment outcome among women who had a positive CT or NG diagnosis at enrolment

Among 193 women who received an on-site GeneXpert testing with a positive CT/NG diagnosis, 84% (162) received treatment within a week from the day of testing, 12% (23) had delayed treatment, with a median time to treatment of 21 days (IQR: 11 days – 37 days) and 4% (8) did not receive CT/NG treatment during pregnancy, **(**Fig. 2**)**. Among 152 women who received laboratory-based testing with a positive diagnosis, 14% (22) received treatment within a week from the day of testing, 63% (96) had delayed treatment, with a median time to treatment was 36 days (IQR: 16 days – 57 days) and 22% (34) did not receive CT/NG treatment during pregnancy (Fig. 2**)**. Overall, the median time to treatment for 119 women who had a delayed treatment in the study was 28 days (IQR: 15 days – 57 days).

### Follow-up during pregnancy

The median duration of follow-up from study enrolment to pregnancy outcome was 15 (IQR 6 to 23) weeks. Among singleton live births, the median gestational age at delivery was 38 (IQR 36 to 40) weeks. Three-quarters of the women reported initiating oral PrEP either at enrolment or during follow-up with a median time of PrEP use during pregnancy of 13 (IQR 3 to 21) weeks, and only 1% (10/919) of those who initiated PrEP had untreated STIs during pregnancy.

### Pregnancy and birth outcomes

**Fig. 3:**
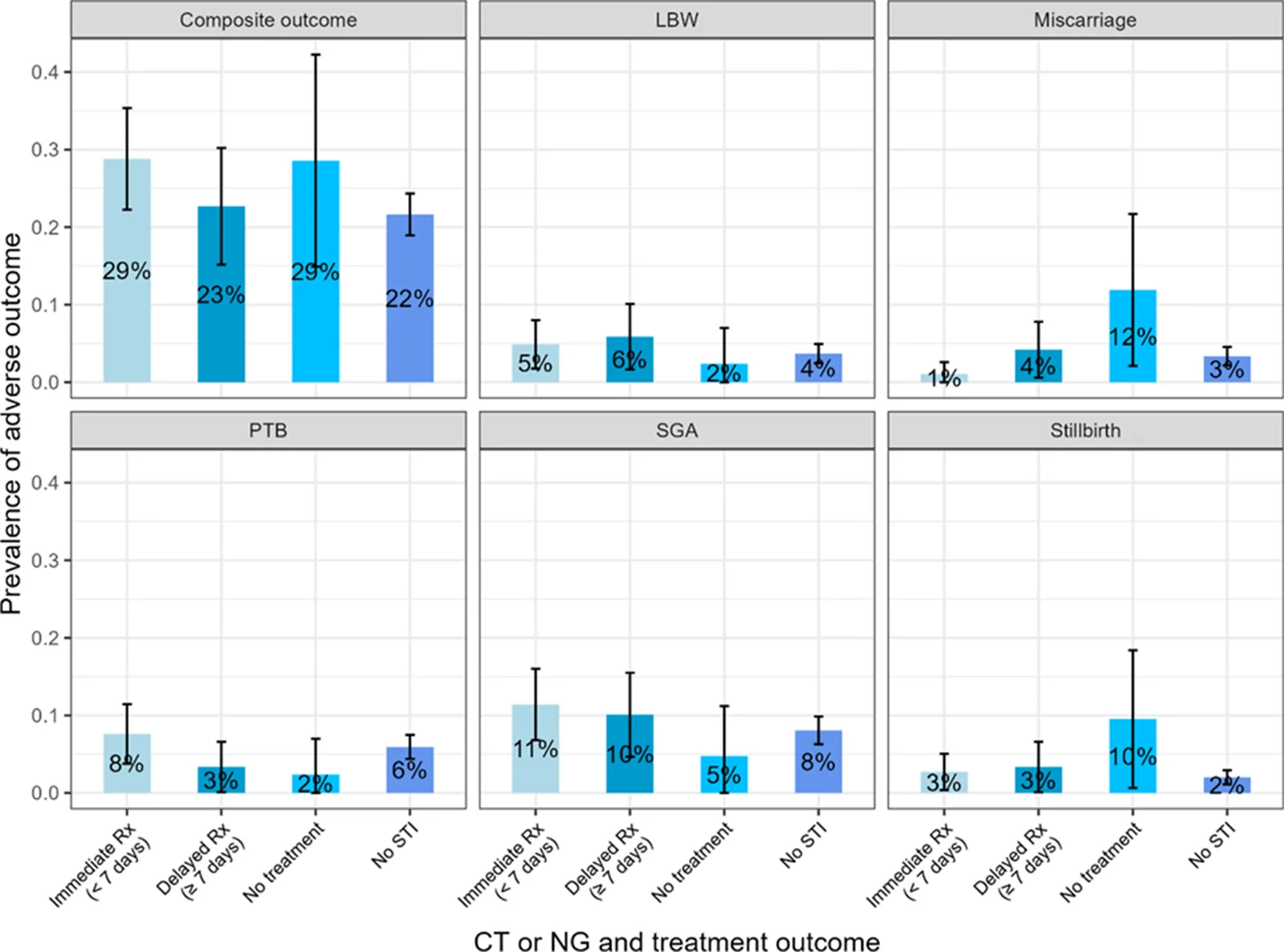
Prevalence of adverse pregnancy and birth outcomes by CT or NG diagnosis and treatment status. Abbreviation: LBW; low birthweight, PTB; preterm birth, SGA; small for gestational age, Rx; treatment, STI; sexually transmitted infection

Among 1237 pregnant women included in the analysis, 23% (285) experienced at least one poor pregnancy outcome. Miscarriage and stillbirth occurred in 3% (42) and 2% (31) of pregnancies, respectively. Overall, 94% of women (1164) had a singleton live birth with median birthweight of 3.2 (IQR 2.8–3.5) kg. Among the singleton live births, 1% (10) resulted in early neonatal death, 6% (72) were PTB, 8% (93) had LBW and 10% (109) were SGA (Table S2). Among the 28% (345/1237) who had CT/NG infection at enrolment, the proportions of any poor pregnancy outcome stratified by timing of CT/NG treatment were 28% (53/184) among women with immediate treatment, 23% (27/119) among those with delayed treatment, and 29% (12/42) among those with no treatment. Among women without CT/NG infection at enrolment (892), 22% (193/892) experienced a poor pregnancy outcome. The timing of CT/NG treatment (immediate, delayed or no treatment) showed borderline difference in the proportion of any poor pregnancy outcome (0.05) (Fig. 3 and Table S2).

In the multivariable analysis, there was no statistically significant differences in the odds of experiencing poor pregnancy outcome between women who had untreated CT/NG or delayed CT/NG treatment compared to those who received immediate treatment for CT/NG (Table 2**)**.

**Table 1:** Baseline characteristics of pregnant women screened for curable STIs in ANC in Cape Town, South Africa (August 2019 - October 2021)

	All women *N* (%)	Any STI (*n*, %)	No STI (*n*, %)	*p*-value
**Sociodemographic characteristics**	1237 (100)	345 (28)	892 (72)	
Maternal age (median, IQR) years	**26 (22–31)**	24 (21–28)	27 (23–32)	< 0.001
16–24 years	**502 (40)**	188 (54)	314 (35)	< 0.001
25–29 years	**368 (30)**	89 (26)	279 (31)	
≥ 30 years	**367 (30)**	68 (20)	299 (34)	
Gravidity				
Multigravida	**820 (66)**	198 (57)	622 (70)	< 0.001
Education level completed				
Secondary or Tertiary	**621 (50)**	184 (53)	437 (49)	0.171
Relationship with father of child				
Not married/not cohabiting	**760 (61)**	246 (71)	514 (58)	< 0.001
Employment status				
Unemployed or attending school/college	**800 (65)**	244 (71)	556 (62)	0.019
Socioeconomic status (SES)				
Low SES	**405 (33)**	111 (32)	294 (33)	0.792
**Clinical characteristics**				
GA at 1st ANC visit (median, IQR) weeks	**22 (15–31)**	22 (16–32)	22 (14–31)	0.343
< 20 weeks @ booking	**506 (41)**	135 (39)	371 (42)	0.429
≥ 20 weeks @ booking	**731 (59)**	210 (61)	521 (58)	
Mode of STI testing				
Laboratory diagnostics	**538 (43)**	152 (44)	386 (43)	0.803
Point-of-care	**699 (57)**	193 (56)	506 (57)	
Calendar period for STI testing				
Pre-COVID-19 lockdown	**360 (29)**	100 (29)	260 (29)	0.955
During-COVID-19 lockdown	**877 (71)**	245 (71)	632 (71)	
**Behavioural characteristics**				
Sexual behavior in the past 3 months				
Vaginal sex	**1204 (97)**	332 (96)	872 (98)	0.149
Number of sex partners				
No sex partner	**33 (3)**	13 (4)	20 (2)	0.040
1 sex partner	**1162 (94)**	326 (94)	836 (94)	
≥2 sex partners	**42 (3)**	6 (2)	36 (4)	
Condom use during sex				
Sometimes/always	**372 (30)**	100 (29)	272 (30)	0.603
Never	**865 (70)**	245 (71)	620 (70)	
Alcohol use prior current pregnancy				
No	**1 165 (94)**	328 (95)	837 (94)	0.397

Abbreviations: IQR, Interquartile range; n, number of participants; GA, gestational age; ANC, antenatal care

Any STI refers to a positive diagnosis for *Chlamydia trachomatis* or *Neisseria gonorrhoeae*

Stratified by each poor pregnancy outcome, there was no difference in the frequency of PTB, LBW and SGA outcomes by timing of treatment between women who had untreated CT/NG or delayed CT/NG treatment compared to those who received immediate treatment for CT/NG (Table 2**)**. In contrast, we observed differences in pregnancy loss by timing of treatment with p-values < 0.01 and 0.02 for miscarriage and stillbirth respectively (Table S2). Among women diagnosed with CT/NG and no treatment during pregnancy, 12% (*n* = 5) and 9% (*n* = 4) experienced a miscarriage or stillbirth respectively. The proportions of miscarriage or stillbirth were lower for women who were diagnosed with CT/NG and received delayed treatment (4%; *n* = 5; and 3%; *n* = 4, respectively), and lowest among women who were diagnosed with CT/NG and received immediate treatment (1%; *n* = 2; and 2%; *n* = 5, respectively). The odds of pregnancy loss (miscarriage and stillbirth) was higher among women who had untreated CT/NG (adjusted odds ratio (aOR) 3,27, 95% CI: 1.09–9.80) compared to those who had immediate CT/NG treatment (Table 2). The odds of pregnancy loss was much higher in women who had untreated CT/NG compared to women who had no CT/NG at study enrolment, (aOR 5.05, 95% CI: 2.23–11.43) (Table S3). There were more neonatal deaths among women who had a diagnosis for CT/NG with immediate treatment (3%, *n* = 5) and women who had no CT/NG at study enrolment (1%, *n* = 5) (*p* = 0.02) (Table S2).

The results were the same in the sensitivity analysis for any poor pregnancy outcome limiting the analysis to women who had gestational age less than 20 weeks at study enrolment. In a sensitivity analysis, restricting the analysis to women who initiated PrEP during pregnancy, there was no association between CT/NG treatment timing and experiencing a poor pregnancy outcome (Table S4). In the sensitivity analysis stratifying delayed treatment group into categories based on time to treatment (8–14 days, 15–28 days, > 28 days), the results did not vary, with no clear trend between increased delayed time to treatment and poor pregnancy outcomes (Table S5).

**Table 2:** Adjusted association between time to treatment of curable STI and pregnancy/birth outcomes in Cape Town, South Africa (2019 - 2021)

Outcome measure	Predictor	N	n	Odds Ratio 95% CI	Adjusted Odds Ratio 95% CI	*p*-value**
Preterm delivery (< 37 weeks)	Immediate treatment	141	13	Ref	Ref	0.10
	Delayed treatment	146	5	0.35 (0.12 - 1.01)	0.37 (0.13 - 1.07)	
	No treatment	33	1	0.30 (0.04 - 2.44)	0.32 (0.04 - 2.96)	
	Immediate treatment	138	11	Ref	Ref	0.61
Low birthweight (< 2500g)	Delayed treatment	143	15	1.35 (0.59 - 3.06)	1.51 (0.63 - 3.60)	
	No treatment	34	2	0.72 (0.15 - 3.43)	0.78 (0.15 - 3.84)	
Small for gestational age (< 10th percentile)	Immediate treatment	133	16	Ref	Ref	0.60
	Delayed treatment	141	17	1.00 (0.48 - 2.08)	1.01 (0.47 - 2.16)	
	No treatment	33	2	0.47 (0.10 - 2.17)	0.50 (0.11 - 2.45)	
Pregnancy loss	Immediate treatment	148	10	Ref	Ref	0.01
	Delayed treatment	155	10	0.95 (0.38 - 2.36)	0.84 (0.31 - 2.27)	
	No treatment	42	9	3.76 (1.41 - 10.01)	3.27 (1.09 - 9.80)	
	Immediate treatment	148	3	Ref	Ref	0.32
Early neonatal death (< 7 days)	Delayed treatment	155	1	0.31 (0.03 - 3.06)	0.28 (0.03 - 2.93)	
	No treatment	42	0	-	-	
Composite adverse outcome	Immediate treatment	148	43	Ref	Ref	0.48
	Delayed treatment	155	36	0.74 (0.44 - 1.23)	0.75 (0.44 - 1.27)	
	No treatment	42	12	0.97 (0.45 - 2.08)	1.01 (0.48 - 2.12)	
Composite adverse outcome*	Immediate treatment	61	19	Ref	Ref	0.16
	Delayed treatment	60	12	0.73 (0.44 - 1.23)	0.69 (0.29 - 1.64)	
	No treatment	14	6	0.97 (0.45 - 2.08)	2.02 (0.57 - 7.11)	

Pregnancy loss includes miscarriage and stillbirth Model adjusted for: maternal age at baseline, calendar period, gravidity, socioeconomic status and relationship statusBinary composite adverse outcome includes at least one of miscarriage, stillbirth, neonatal death, PTB, LBW and SGA

*Model limiting to participants who had gestational age < 20 weeks**p-value using Wald test

## Discussion

In this prospective observational study, we found a high prevalence of CT and NG at enrolment in pregnant women without HIV. In this study, women who received a positive diagnosis using on-site GeneXpert STI testing were more likely to receive immediate treatment than those diagnosed using laboratory-based testing, suggesting that on-site GeneXpert STI testing facilitated immediate treatment compared with laboratory-based testing. Nearly a quarter of the women experienced poor pregnancy outcome. The likelihood of a poor pregnancy outcome among pregnant women with a positive CT/NG diagnosis varied by timing of treatment, showing a borderline difference between those who received immediate, delayed, or no treatment. Pregnant women with untreated CT and/or NG had a 4-fold increased odds of experiencing a pregnancy loss (miscarriage or stillbirth). We did not find an association between either a positive diagnosis or time to treatment for CT or NG and any poor pregnancy outcome.

Our finding that on-site GeneXpert STI testing facilitated immediate treatment compared with laboratory-based testing is consistent with findings from previous studies in South Africa [25, 34]. In the HVTN702 HIV vaccine trial, women diagnosed with CT or NG via POC testing started treatment approximately 39 times faster than those diagnosed through laboratory-based testing. However, it is important to note that COVID-19 restrictions, including lockdowns, reduced clinic visits, and strain on laboratory services, may have further exacerbated delays in results return and treatment initiation for those relying on laboratory-based STI testing during the study period, highlighting additional advantage of utilizing POC testing with potential for same day treatment.

In our previous study in a different cohort [35], diagnosis and treatment of CT or NG at first ANC were each significantly associated with increased risk of having any adverse pregnancy outcome. We were unable to evaluate impact of time to treatment on the adverse pregnancy and birth outcomes. In this analysis, the sample is relatively large and the time to treatment varied due to a transition from on-site GeneXpert testing to laboratory-based STI testing during the study period. However, the lack of association between the time to treatment for CT or NG and any poor pregnancy outcome may be attributed to the low incidence of adverse events during pregnancy. Also, because women in this cohort were not randomized to on-site GeneXpert or laboratory-based testing, and these tests were conducted over different time periods, we therefore, cannot rule out the possibility of confounding in our estimates of the effects of timing of STI treatment during pregnancy on pregnancy and birth outcomes. For example, in women who received laboratory testing, those presenting with more severe symptoms or STI related complications were more likely to receive immediate treatment based on syndromic management, resulting in an overrepresentation of women with severe infections in the immediate treatment group. Therefore, our study findings do not disprove a possible effect of STI and time to treatment during pregnancy on adverse pregnancy outcomes.

Additionally, neither diagnosis nor time to treatment of CT or NG significantly impacted the PTB and SGA outcomes. In the WANTAIM trial, POC testing and treatment for curable STIs did not significantly reduce rates of PTB or LBW compared to syndromic management [19]. Notably, there was reduction in CT cases in the control group, likely due to high uptake of sulphadoxine-pyrimethamine for malaria prophylaxis and may have impacted overall trial outcomes. It is also worth noting that the trial had a very low prevalence of confirmed women living with HIV (< 1%), closely mirroring our cohort of women without HIV [19]. Considering the uncertainty surrounding the benefits of aetiological testing and treatment for curable STIs during pregnancy on adverse outcomes, there is a clear need for larger randomized trials. These trials should aim to more accurately examine and establish any causal relationships between STI diagnosis, timing of treatment, and adverse pregnancy outcomes.

In our study, women with untreated CT and/or NG during pregnancy had a 4-fold increased odds of experiencing a miscarriage or stillbirth. Adverse outcomes associated with infection in the first trimester are plausible as the first trimester is the key period in which vital organs of the fetus develop [4, 36]. In our analysis, we combined miscarriage and stillbirth as our pregnancy loss measure and more than 41% of the participants had gestational age of less than 20 weeks at first ANC visit (still eligible for a miscarriage outcome). Despite this, our study likely underestimates pregnancy loss due to unrecorded miscarriages or stillbirths resulting from delayed ANC booking. These results should be interpreted with caution due to potential reverse causality. While untreated STIs may increase miscarriage or stillbirth risk, women who experience pregnancy loss may be less likely to return to the clinic for STI treatment, making the direction of cause and effect uncertain. It is important to note that the proportions of both miscarriage and stillbirth observed in this study were comparable to national estimates for South Africa [37] and are notably higher compared to high-income countries [38], highlighting the need for targeted health interventions to address these outcomes in South Africa and similar regions. These results emphasize the importance of timely intervention for curable STIs in reducing the risk of miscarriage and stillbirth. A potential explanation for the observed association between delayed treatment and pregnancy loss relates to the timing of infection relative to diagnosis and treatment. In this study, we were unable to account for the temporal dynamics of STI acquisition, including the interval between infection onset, detection, and initiation of treatment. As a result, time to treatment may not fully capture the biologically relevant window during which infection exerts its effects on pregnancy. Given that many women likely acquire STIs before pregnancy, testing early provides an opportunity to detect and treat infections before they can progress or cause complications during pregnancy. Early neonatal deaths occurred in less than 1% of participants in this study and were evenly distributed between women with STIs who received immediate treatment (50%) and those without STIs (50%). While the association appears significant, it is not clear why this is the case, and other confounding factors may have contributed to neonatal deaths in these groups, warranting further investigation.

In a sensitivity analysis restricting the analysis to women who initiated oral PrEP during pregnancy, our results showed no association between the occurrence of poor pregnancy outcome and time to STI treatment. This finding could be explained by high proportion of women in this subgroup who received STI treatment during pregnancy, potentially mitigating the impact of any treatment delays. Another possibility is that women using PrEP are self-identified as being at high risk for HIV and may also have been at increased risk for other STIs, which could have influenced the observed finding. Our previous analysis of this cohort showed no difference in pregnancy outcomes by PrEP exposure using both self-report and objective measure of intracellular tenofovir diphosphate [39]. However, further research would be needed to better understand the interaction between PrEP use, STI status and treatment timing, and pregnancy-related outcomes.

In this study, curable STIs were diagnosed using on-site GeneXpert testing or laboratory-based PCR testing. While on-site GeneXpert testing offers an efficient diagnostic solution, the 90-minute turnaround time for results remains a potential barrier to ensuring timely treatment. Based on recent price listing, the estimated cost of public health diagnostic testing of CT or NG in South Africa is approximately $32 at the central laboratory, whereas point-of-care GeneXpert testing is estimated at $56 per test [40, 41]. However, there is need to develop cost-effective, affordable POC diagnostic tests that are readily accessible, which can be seamlessly integrated into routine antenatal care. We acknowledge that the training requirements and operational costs associated with implementing screening for curable STIs using diagnostic tests may pose significant challenges for healthcare facilities, particularly in resource-constrained settings where access to laboratory infrastructure is limited. However, South Africa’s 2011 national rollout of GeneXpert for tuberculosis (TB) diagnosis [42, 43], resulted in the establishment of a network laboratories with GeneXpert instruments across the country. While primarily used for TB, GeneXpert platforms can also be utilized for the detection of some curable STIs, presenting an opportunity to leverage existing infrastructure for expanded STI testing, though potential limitations and the need for further investment in capacity and consumables would need to be carefully considered.

Our study has limitations that should be acknowledged. Information on STI symptoms was not collected to evaluate proportion of women who could have received same day treatment through syndromic management. However, previous studies have shown that most of the STI cases during pregnancy are usually asymptomatic [14–16]. We did not have information on syphilis screening and treatment which could have an impact on pregnancy and birth outcomes. Another limitation was the lack of data on partner treatment and reinfection during pregnancy. Although aetiologic STI screening may support targeted partner notification and reduce reinfection risk, we could not assess these strategies. This may have affected treatment effectiveness and outcomes. Future studies should evaluate partner management, reinfection, and repeat testing. Additionally, while unmeasured confounding remains a limitation inherent to observational studies, we employed rigorous study design and analysis techniques to mitigate its potential impact. It is also important to acknowledge that our findings establish associations rather than definitive causal relationships.

## Conclusion

In this cohort study of pregnant women without HIV, we observed a high prevalence of CT and NG infections at enrolment. On-site testing showed potential advantages, with a significantly higher proportion of women receiving immediate treatment compared to those diagnosed through laboratory-based testing. However, adverse pregnancy outcomes were not conclusively associated with diagnosis or timing of treatment for these STIs. Untreated infections were associated with increased risk of miscarriage or stillbirth, underlining the importance of timely intervention. Our findings suggest that integrating routine POC STI testing and rapid treatment in antenatal care could play a vital role in reducing pregnancy complications, but larger trials are necessary to clarify these associations.

## Supplementary Information

Below is the link to the electronic supplementary material.

Supplementary Material 1

## Data Availability

The datasets used and analysed during this current study are available from corresponding author on request.

## Abbreviations

ANC: Antenatal clinic
CT: Chlamydia trachomatis
GA: Gestational age
LMICs: low- and middle-income countries
NG: Neisseria gonorrhoeae
STIs: Sexually transmitted infections
