# Supplementary material for: Impact of antenatal screening for Chlamydia and Gonorrhea on pregnancy outcomes in South African women
Source: BMC Infect Dis. 2026 Jul 21;26:1559. doi: 10.1186/s12879-026-13967-3 (PMC13505017; doi:10.1186/s12879-026-13967-3)
Supplement: Supplementary file 1 [file 12879_2026_13967_MOESM1_ESM.docx]

| **Supplementary Table S1. Baseline characteristics of pregnant women by type of CT/NG screening** | | | | |
| --- | --- | --- | --- | --- |
|  | **All women N (%)** | **Lab-based tests** | **Gene Xpert tests** | **p-value** |
| **Sociodemographic characteristics** | **1237 (100)** | **538 (43)** | **699 (57)** |  |
| Maternal age (median, IQR) years | **26 (22 - 31)** | 26 (22 - 31) | 26 (22 - 31) | 0.285 |
| 16 - 24 years | **502 (40)** | 213 (40) | 289 (41) | 0.290 |
| 25 - 29 years | **368 (30)** | 153 (28) | 215 (31) |  |
| ≥ 30 years | **367 (30)** | 172 (32) | 195 (28) |  |
| Gravidity |  |  |  |  |
| Multigravida | **820 (66)** | 365 (67) | 455 (65) | 0.524 |
| Education level completed |  |  |  |  |
| Secondary or Tertiary | **621 (50)** | 265 (49) | 356 (51) | 0.560 |
| Relationship with father of child |  |  |  |  |
| Not married/not cohabiting | **759 (61)** | 320 (59) | 439 (63) | 0.250 |
| Employment status |  |  |  |  |
| Unemployed or school/college | **800 (65)** | 369 (68) | 431 (62) | 0.006 |
| Socioeconomic status (SES) |  |  |  |  |
| Low SES | **405 (33)** | 194 (36) | 211 (30) | 0.029 |
| **Clinical characteristics** |  |  |  |  |
| GA at 1st ANC visit (median, IQR) weeks | **22 (15 - 31)** | 23 (15 - 33) | 21 (14 - 30) | 0.075 |
| < 20 weeks @ booking | **506 (41)** | 209 (39) | 297 (42) | 0.196 |
| ≥ 20 weeks @ booking | **731 (59)** | 329 (61) | 402 (58) |  |
| Calendar period for STI testing |  |  |  |  |
| Pre-COVID-19 lockdown | **360 (29)** | 0 (0) | 360 (52) | - |
| During-COVID-19 lockdown | **877 (71)** | 538 (100) | 339 (48) |  |
| **Behavioural characteristics** |  |  |  |  |
| Sexual behaviour in the past 3 months |  |  |  |  |
| Vaginal sex | **1204 (97)** | 524 (97) | 680 (97) | 0.900 |
| Number of sex partners |  |  |  |  |
| No sex partner | **33 (3)** | 14 (3) | 19 (3) | 0.762 |
| 1 sex partner | **1162 (94)** | 508 (94) | 654 (93) |  |
| ≥2 sex partners | **42 (3)** | 16 (3) | 26 (4) |  |
| Condom use during sex |  |  |  |  |
| Sometimes/always | **372 (30)** | 156 (29) | 216 (30) | 0.468 |
| Never | **865 (70)** | 382 (71) | 483 (70) |  |
| Alcohol use prior current pregnancy |  |  |  |  |
| No | **1 165 (94)** | 504 (94) | 661 (94) | 0.512 |
| Abbreviations: IQR, Interquartile range; n, number of participants; GA, gestational age; ANC, antenatal care | | | | |
| Any STI refers to a positive diagnosis for *Chlamydia trachomatis* or *Neisseria gonorrhoeae* | | | | |

| **Supplementary Table S2: Pregnancy and birth outcomes by time to treatment of curable STIs during pregnancy Cape Town, South Africa (2019 - 2021** | | | | | | | | | | | |
| --- | --- | --- | --- | --- | --- | --- | --- | --- | --- | --- | --- |
|  | **All women** | | **STI, immediate treatment**  **n (%)** | | | | **STI, delayed treatment**  **n (%)** | | **STI, untreated**  **n (%)** | | **No STI n (%)** |
| **Characteristic** | **1237 (100)** | | **184 (15)** | | | | **119 (10)** | | **42 (3)** | | **892 (72)** |
| **Pregnancy loss** |  | |  | | | |  | |  | |  |
| Total pregnancy loss | **73 (6)** | | **7 (4 )** | | | | **9 (7 )** | | **9 (21)** | | **48 (5)** |
| Miscarriage GA ≤ 20 weeks | 42 (3) | | 2 (1) | | | | 5 (4) | | 5 (12) | | 30 (3) |
| Stillbirth GA > 20 weeks | 31 (2) | | 5 (2) | | | | 4 (3) | | 4 (9) | | 18 (2) |
| **Singleton live birth outcomes** |  | |  | | | |  | |  | |  |
| Total live births | **1164 (94)** | | **177 (96)** | | | | **110 (93)** | | **33 (79)** | | **844 (95)** |
| Full term delivery GA ≥ 37 weeks | 1089 (87) | | 161 (86) | | | | 107 (89) | | 32 (76) | | 789 (88) |
| Preterm delivery GA < 37 weeks | 72 (6) | | 14 (7) | | | | 4 (3) | | 1 (2) | | 53 (6) |
| Neonatal death* | 10 (1) | | 5 (3) | | | | 0 (0) | | 0 (0) | | 5 (1) |
| LBW< 2500g | 93 (8) | | 15 (9) | | | | 11 (10) | | 2 (6) | | 65 (8) |
| LBW< 2500g ** | 50 (5) | | 9 (6) | | | | 7 (7) | | 1 (3) | | 33 (4) |
| SGA < 10th percentile* | 107 (10) | | 21 (12) | | | | 12 (11) | | 2 (6) | | 72 (9) |
| SGA - full term GA ≥ 37 weeks | 99 (9) | | 21 (14) | | | | 12 (11) | | 2 (6) | | 64 (8) |
| SGA - preterm GA < 37 weeks | 8 (12) | | 0 (0) | | | | 0 (0) | | 0 (0) | | 8 (16) |
| AGA > 10th percentile and < 90th percentile * | 890 (79) | | 123 (73) | | | | 85 (78) | | 29 (87) | | 653 (80) |
| AGA - full term GA ≥ 37 weeks | 841 (79) | | 114 (73) | | | | 81 (77) | | 28 (87) | | 618 (81) |
| AGA - preterm GA < 37 weeks | 49 (73) | | 9 (69) | | | | 4 (100) | | 1 (100) | | 35 (72) |
| LGA > 90th percentile* | 128(11) | | 24 (14) | | | | 12 (11) | | 2 (6) | | 90 (11) |
| LGA - full term GA ≥ 37 weeks | 118 (11) | | 20 (13) | | | | 12 (11) | | 2 (6) | | 84 (11) |
| LGA - preterm GA < 37 weeks | 10 (14) | | 4 (31) | | | | 0 (0) | | 0 (0) | | 6 (11) |
| Birth weight (median, IQR) grams (**n=1160)** | 3.2 (2.9 - 3.5) | | 3.1 (2.8 - 3.5) | | | | 3.2 (2.8 - 3.5) | | 3.2 (2.8 - 3.5) | | 3.2 (2.9 - 3.5) |
| **Composite adverse outcome(N = 1237)** |  | |  | | | |  | |  | |  |
| Adverse outcome | 285 (23) | | 53 (29) | | | | 27 (23) | | 12 (29) | | 193 (22) |
| No adverse outcome | 952 (77) | | 131 (71) | | | | 92 (77) | | 30 (71) | | 699 (78) |
| Abbreviations: AGA, appropriate for gestational age; GA, gestational age; IQR, Interquartile range; LGA, large for gestational age; LBW, low birthweight; SGA, small for gestational age | | | | | | | | | | | |
| N=1237, excluding termination of pregnancy, multiple births and maternal HIV seroconversion | | | | | | | | | | | |
| * among all live births excluding multiple births and ** among full term births excluding multiple births | | | | | | | | | | | |
| **Supplementary Table S3: Adjusted association between curable STI diagnosis and treatment at study enrolment and adverse birth outcomes (2019 - 2021)** | | | | | | | | | | | |
| Outcome measure | | Predictor | | N | n | Odds Ratio 95% CI | | Adjusted Odds Ratio 95% CI | | p-value ** | |
| Preterm delivery (< 37 weeks) | | No STI | | 844 | 53 | Ref | | Ref | | 0.45 | |
|  |  | Immediate treatment | | 177 | 14 | 1.28 (0.69 - 2.36) | | 1.36 (0.74 - 2.51) | |  | |
|  |  | Delayed treatment | | 110 | 4 | 0.56 (0.19 - 1.58) | | 0.57 (0.20 - 1.63) | |  | |
|  |  | No treatment | | 33 | 1 | 0.46 (0.06 - 3.49) | | 0.48 (0.06 - 3.72) | |  | |
|  | | No STI | | 838 | 65 | Ref | | Ref | | 0.78 | |
| Low birthweight (< 2500g) | | Immediate treatment | | 173 | 15 | 1.12 (0.62 - 2.03) | | 1.05 (0.57 - 1.91) | |  | |
|  | | Delayed treatment | | 108 | 11 | 1.34 (0.68 - 2.64) | | 1.34 (0.66 - 2.68) | |  | |
|  | | No treatment | | 34 | 2 | 0.74 (0.17 - 3.17) | | 0.75 (0.17 - 3.26) | |  | |
| Small for gestational age (< 10th percentile) | | No STI | | 812 | 72 | Ref | | Ref | | 0.39 | |
|  |  | Immediate treatment | | 166 | 21 | 1.48 (0.88 - 2.49) | | 1.31 (0.78 - 2.20) | |  | |
|  |  | Delayed treatment | | 108 | 12 | 1.28 (0.67 - 2.45) | | 1.12 (0.57 - 2.18) | |  | |
|  |  | No treatment | | 33 | 2 | 0.66 (0.15 - 2.83) | | 0.59 (0.13 - 2.55) | |  | |
| Pregnant loss | | No STI | | 892 | 53 | Ref | | Ref | | 0.003 | |
|  |  | Immediate treatment | | 184 | 11 | 1.00 (0.51 - 1.96) | | 0.95 (0.47 – 1.91) | |  | |
|  |  | Delayed treatment | | 119 | 9 | 1.29 (0.62 - 2.70) | | 1.32 (0.63 - 2.75) | |  | |
|  |  | No treatment | | 42 | 9 | **4.31 (1.96 - 9.49)** | | **5.05 (2.23 - 11.43)** | |  | |
| Early neonatal death (< 7 days) | | No STI | | 892 | 5 | Ref | | Ref | | 0.04 | |
|  |  | Immediate treatment | | 184 | 4 | **3.94 (1.04 - 14.83)** | | 3.11 (0.92 - 10.53) | |  | |
|  |  | Delayed treatment | | 119 | 0 | **-** | | **-** | |  | |
|  |  | No treatment | | 42 | 0 | **-** | | - | |  | |
| Composite adverse outcome | | No STI | | 892 | 193 | Ref | | Ref | | 0.21 | |
|  |  | Immediate treatment | | 184 | 52 | 1.42 (0.99 - 2.04) | | 1.34 (0.93 - 1.92) | |  | |
|  |  | Delayed treatment | | 119 | 27 | 1.06 (0.67 - 1.68) | | 1.01 (0.63 - 1.60) | |  | |
|  |  | No treatment | | 42 | 12 | 1.44 (0.73 - 2.88) | | 1.39 (0.72 - 2.72) | |  | |
| Composite adverse outcome* | | No STI | | 371 | 97 | Ref | | Ref | | 0.41 | |
|  |  | Immediate treatment | | 77 | 22 | 1.13 (0.65 - 1.95) | | 0.98 (0.57 - 1.70) | |  | |
|  |  | Delayed treatment | | 44 | 9 | 0.72 (0.33 - 1.57) | | 0.67 (0.32 - 1.42) | |  | |
|  |  | No treatment | | 14 | 6 | 2.12 (0.71 - 6.27) | | 1.98(0.68 - 5.72) | |  | |
| STI exposure defined as positive diagnosis of curable STI at study enrolment | | | | | | | | | |  | |
| Pregnancy loss includes miscarriage and stillbirth | | | | | | | | | |  | |
| Model adjusted for: maternal age, calendar period, gravidity, socioeconomic and marital status | | | | | | | | | | | |
| *Model limiting to participants who had gestational age < 20 weeks | | | | | | | | | | | |
| **p-value using Wald test | | | | | | | | | | | |

| **Supplementary Table S4: Adjusted association between time to treatment of curable STI and pregnancy/birth outcomes among women who initiated HIV PrEP during pregnancy ( 2019 - 2021)** | | | | | | |
| --- | --- | --- | --- | --- | --- | --- |
| Outcome measure | Predictor | N | n | Odds Ratio 95% CI | Adjusted Odds Ratio 95% CI | p-value** |
| Preterm delivery (< 37 weeks) | Immediate treatment | 118 | 11 | Ref | Ref | 0.12 |
|  | Delayed treatment | 121 | 5 | 0.42 (0.14 - 1.25) | 0.43 (0.14 - 1.31) |  |
|  | No treatment | 9 | 0 | **-** | **-** |  |
| Low birthweight (<2500g) | Immediate treatment | 116 | 9 | Ref | Ref | 0.41 |
|  | Delayed treatment | 119 | 13 | 1.45 (0.59 - 3.56) | 1.50 (0.54 - 4.13) |  |
|  | No treatment | 9 | 0 | **-** | **-** |  |
| Small for gestational age (< 10th percentile) | Immediate treatment | 112 | 13 | Ref | Ref | 0.96 |
|  | Delayed treatment | 118 | 15 | 1.11 (0.50 - 2.45) | 1.15 (0.50 - 2.65) |  |
|  | No treatment | 9 | 1 | 0.95 (0.11 - 8.27) | 0.98 (0.12 - 7.53) |  |
| Pregnant loss | Immediate treatment | 121 | 6 | Ref | Ref | 0.80 |
|  | Delayed treatment | 128 | 7 | 1.10 (0.36 - 3.40) | 1.03 (0.30 - 3.46) |  |
|  | No treatment | 10 | 1 | 2.12 (0.23 - 19.75) | 2.18 (0.20 - 23.75) |  |
| Early neonatal death | Immediate treatment | 121 | 3 | Ref | Ref | 0.34 |
|  | Delayed treatment | 128 | 0 | 0.33 (0.03 - 3.28) | 0.33 (0.03 - 3.54) |  |
|  | No treatment | 10 | 0 | **-** | **-** |  |
| Composite adverse outcome | Immediate treatment | 121 | 32 | Ref | Ref | 0.81 |
|  | Delayed treatment | 128 | 30 | 0.85 (0.48 - 1.51) | 0.92 (0.51 - 1.65) |  |
|  | No treatment | 10 | 2 | 0.69 (0.13 - 3.45) | 0.70 (0.15 - 3.27) |  |
| Composite adverse outcome* | Immediate treatment | 50 | 16 | Ref | Ref | 0.16 |
|  | Delayed treatment | 51 | 10 | 0.73 (0.44 - 1.23) | 0.69 (0.29 - 1.64) |  |
|  | No treatment | 2 | 1 | 0.97 (0.45 - 2.08) | 2.02 (0.57 - 7.11) |  |
| Pregnancy loss includes miscarriage and stillbirth | | | | | | |
| Model adjusted for: maternal age at baseline, duration on PrEP until birth, gravidity socioeconomic status and marital status | | | | | | |
| Composite adverse outcome includes all miscarriage, stillbirth, neonatal death and SGA | | | | | | |
| *Model limiting to participants who had gestational age < 20 weeks | | | | | | |
| **p-value using Wald test | | | | | | |

| **Supplementary Table S5: Adjusted association between time to treatment of curable STI and pregnancy/birth outcomes ( 2019 - 2021)** | | | | | | |
| --- | --- | --- | --- | --- | --- | --- |
| Outcome measure | Predictor | N | n | Odds Ratio 95% CI | Adjusted Odds Ratio 95% CI | p-value** |
| Preterm delivery (< 37 weeks) | Immediate treatment | 177 | 14 | Ref | Ref | 0.51 |
|  | Delayed 8–14 days | 27 | 2 | 0.93 (0.19 - 4.35) | 1.16 (0.25 - 5.38) |  |
|  | Delayed 15–28 days | 27 | 1 | 0.45 (0.06 - 3.56) | 0.42 (0.05 - 3.40) |  |
|  | Delayed >28 days | 56 | 1 | 0.21 (0.03 - 1.65) | 0.23 (0.03 - 1.71) |  |
|  | No treatment | 33 | 1 | 0.36 (0.05 - 2.87) | 0.38 (0.04 - 3.50) |  |
| Low birthweight (<2500g) | Immediate treatment | 173 | 15 | Ref | Ref | 0.52 |
|  | Delayed 8–14 days | 27 | 4 | 1.83 (0.56 - 6.01) | 2.51 (0.73 - 8.63) |  |
|  | Delayed 15–28 days | 25 | 1 | 0.44 (0.05 - 3.48) | 0.49 (0.05 - 4.05) |  |
|  | Delayed >28 days | 56 | 6 | 1.26 (0.46 - 3.43) | 1.26 (0.45 - 3.59) |  |
|  | No treatment | 34 | 2 | 0.66 (0.14 - 3.03) | 0.74 (0.15 - 3.51) |  |
| Small for gestational age (< 10th percentile) | Immediate treatment | 166 | 21 | Ref | Ref | 0.96 |
|  | Delayed 8–14 days | 27 | 3 | 0.86 (0.23 - 3.12) | 0.83 (0.23 - 3.05) |  |
|  | Delayed 15–28 days | 25 | 3 | 0.94 (0.25 - 3.43) | 0.96 (0.28 - 3.26) |  |
|  | Delayed >28 days | 56 | 6 | 0.83 (0.31 - 2.17) | 0.74 (0.26 - 2.10) |  |
|  | No treatment | 33 | 2 | 0.44 (0.09 - 2.00) | 0.48 (0.10 - 2.28) |  |
| Pregnant loss | Immediate treatment | 184 | 11 | Ref | Ref | 0.33 |
|  | Delayed 8–14 days | 28 | 0 | **-** | **-** |  |
|  | Delayed 15–28 days | 28 | 2 | 0.58 (0.07 - 4.71) | 0.60 (0.05 - 7.03) |  |
|  | Delayed >28 days | 57 | 1 | 0.83 (0.31 - 2.23) | 0.69 (0.08 - 5.49) |  |
|  | No treatment | 37 | 4 | 1.90 (0.57 - 6.36) | 2.11 (0.46 - 9.59) |  |
| Early neonatal death | Immediate treatment | 184 | 3 | Ref | Ref | 0.46 |
|  | Delayed 8–14 days | 29 | 1 | 0.28 (0.07 - 5.31) | 0.37 (0.12 - 6.57) |  |
|  | Delayed 15–28 days | 31 | 0 | **-** | **-** |  |
|  | Delayed >28 days | 59 | 0 | **-** | **-** |  |
|  | No treatment | 42 | 0 | **-** | **-** |  |
| Composite adverse outcome | Immediate treatment | 184 | 52 | Ref | Ref | 0.30 |
|  | Delayed 8–14 days | 29 | 6 | 0.69 (0.26 - 1.80) | 0.87 (0.31 - 2.41) |  |
|  | Delayed 15–28 days | 31 | 7 | 0.84 (0.33 - 2.11) | 0.87 (0.34 - 2.19) |  |
|  | Delayed >28 days | 59 | 8 | 0.91 (0.18 - 1.80) | 0.91 (0.18 - 1.85) |  |
|  | No treatment | 42 | 7 | 0.59 (0.24 - 1.43) | 0.64 (0.26 - 1.59) |  |
| Composite adverse outcome* | Immediate treatment | 77 | 22 | Ref | Ref | 0.68 |
|  | Delayed 8–14 days | 8 | 1 | 0.36 (0.04 - 3.10) | 0.68 (0.08 - 5.78) |  |
|  | Delayed 15–28 days | 10 | 2 | 0.62 (0.12 - 3.20) | 0.71 (0.10 - 4.62) |  |
|  | Delayed >28 days | 23 | 3 | 0.37 (0.10 - 1.39) | 0.39 (0.10 - 1.46) |  |
|  | No treatment | 11 | 3 | 0.94 (0.22 - 3.88) | 1.27 (0.24 - 6.65) |  |
| Pregnancy loss includes miscarriage and stillbirth | | | | | | |
| Model adjusted for: maternal age at baseline, duration on PrEP until birth, gravidity socioeconomic status and marital status | | | | | | |
| Composite adverse outcome includes all miscarriage, stillbirth, neonatal death and SGA | | | | | | |
| *Model limiting to participants who had gestational age < 20 weeks | | | | | | |
| **p-value using Wald test | | | | | | |
